# Functional characterization of human recessive *DIS3* variants in premature ovarian insufficiency^†^

**DOI:** 10.1093/biolre/ioae148

**Published:** 2024-10-14

**Authors:** Brianna L Kline, Nicole A Siddall, Fernando Wijaya, Catherine J Stuart, Luisa Orlando, Shabnam Bakhshalizadeh, Fateme Afkhami, Katrina M Bell, Sylvie Jaillard, Gorjana Robevska, Jocelyn A van den Bergen, Shirin Shahbazi, Ambro van Hoof, Katie L Ayers, Gary R Hime, Andrew H Sinclair, Elena J Tucker

**Affiliations:** Murdoch Children's Research Institute, Royal Children's Hospital, 50 Flemington Rd, Parkville VIC 3052, Melbourne, Australia; Department of Paediatrics, The University of Melbourne, Grattan Street, Parkville, VIC 3010, Melbourne, Australia; Department of Anatomy and Physiology, The University of Melbourne, Grattan Street, Parkville, VIC 3010, Melbourne, Australia; Department of Anatomy and Physiology, The University of Melbourne, Grattan Street, Parkville, VIC 3010, Melbourne, Australia; Department of Microbiology and Molecular Genetics, University of Texas Health Science Center at Houston, 7000 Fannin, Suite 1706, Houston, TX 77030, USA; Department of Microbiology and Molecular Genetics, University of Texas Health Science Center at Houston, 7000 Fannin, Suite 1706, Houston, TX 77030, USA; Murdoch Children's Research Institute, Royal Children's Hospital, 50 Flemington Rd, Parkville VIC 3052, Melbourne, Australia; Department of Paediatrics, The University of Melbourne, Grattan Street, Parkville, VIC 3010, Melbourne, Australia; Department of Medical Genetics, Faculty of Medical Sciences, Tarbiat Modares University, Tehran Province, Tehran, Jalal Al Ahmad St, P9CJ+HC9, Iran; Murdoch Children's Research Institute, Royal Children's Hospital, 50 Flemington Rd, Parkville VIC 3052, Melbourne, Australia; Murdoch Children's Research Institute, Royal Children's Hospital, 50 Flemington Rd, Parkville VIC 3052, Melbourne, Australia; INSERM, Institut de Recherche en Santé, Environement et Travail, University of Rennes, 9 Av. du Professeur Léon Bernard, 35000, Rennes, France; CHU Rennes, Service de Cytogénétique et Biologie Cellulaire, 2 rue Henri Le Guilloux, 35033 Rennes CEDEX 9F-35033, France; Murdoch Children's Research Institute, Royal Children's Hospital, 50 Flemington Rd, Parkville VIC 3052, Melbourne, Australia; Murdoch Children's Research Institute, Royal Children's Hospital, 50 Flemington Rd, Parkville VIC 3052, Melbourne, Australia; Department of Medical Genetics, Faculty of Medical Sciences, Tarbiat Modares University, Tehran Province, Tehran, Jalal Al Ahmad St, P9CJ+HC9, Iran; Department of Microbiology and Molecular Genetics, University of Texas Health Science Center at Houston, 7000 Fannin, Suite 1706, Houston, TX 77030, USA; Murdoch Children's Research Institute, Royal Children's Hospital, 50 Flemington Rd, Parkville VIC 3052, Melbourne, Australia; Department of Paediatrics, The University of Melbourne, Grattan Street, Parkville, VIC 3010, Melbourne, Australia; Department of Anatomy and Physiology, The University of Melbourne, Grattan Street, Parkville, VIC 3010, Melbourne, Australia; Murdoch Children's Research Institute, Royal Children's Hospital, 50 Flemington Rd, Parkville VIC 3052, Melbourne, Australia; Department of Paediatrics, The University of Melbourne, Grattan Street, Parkville, VIC 3010, Melbourne, Australia; Murdoch Children's Research Institute, Royal Children's Hospital, 50 Flemington Rd, Parkville VIC 3052, Melbourne, Australia; Department of Paediatrics, The University of Melbourne, Grattan Street, Parkville, VIC 3010, Melbourne, Australia

**Keywords:** premature ovarian insufficiency, exosome, DIS3, infertility, RNA, Drosophila, WES, genetics

## Abstract

Premature ovarian insufficiency (POI) is characterized by the loss or complete absence of ovarian activity in women under the age of 40. Clinical presentation of POI varies with phenotypic severity ranging from premature loss of menses to complete gonadal dysgenesis. POI is genetically heterogeneous with >100 causative gene variants identified thus far. The etiology of POI varies from syndromic, idiopathic, monogenic to autoimmune causes the condition. Genetic diagnoses are beneficial to those impacted by POI as it allows for improved clinical management and fertility preservation. Identifying novel variants in candidate POI genes, however, is insufficient to make clinical diagnoses. The impact of missense variants can be predicted using bioinformatic algorithms but computational approaches have limitations and can generate false positive and false negative predictions. Functional characterization of missense variants, is therefore imperative, particularly for genes lacking a well-established genotype:phenotype correlation. Here we used whole-exome sequencing (WES) to identify the first case of a homozygous missense variant in *DIS3* (c.2320C > T; p.His774Tyr) a critical component of the RNA exosome in a POI patient. This adds to the previously described compound heterozygous patient. We perform the first functional characterization of a human POI-associated *DIS3* variant. A slight defect in mitotic growth was caused by the variant in a *Saccharomyces cerevisiae* model. Transgenic rescue of *Dis3* knockdown in *Drosophila melanogaster* with human *DIS3* carrying the patient variant led to aberrant ovarian development and egg chamber degeneration. This supports a potential deleterious impact of the human c.2320C > T; p.His774Tyr variant.

## Teaser

**Summary Sentence**

*DIS3* variant identified in a patient with premature ovarian insufficiency has reduced capacity to rescue *Drosophila* ovarian *Dis3* knockdown compared to wildtype, suggesting the variant is hypomorphic and the RNA exosome is critical for ovarian function.

## Graphical Abstract

**Graphical Abstract:**
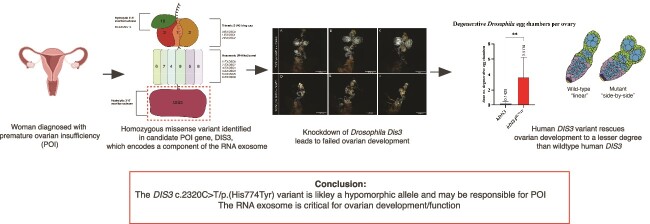

## Introduction

Premature ovarian insufficiency (POI), characterized by the loss or complete absence of ovarian activity, is a common cause of female infertility affecting ~1-3.7% of women under the age of 40 [1–3]. Clinical presentation can vary from premature loss of menses (secondary amenorrhea) or failure to enter menarche (primary amenorrhea) associated with complete absence of gonadal structures (streak gonads). POI is diagnosed by elevated (>25 mlU/ml) Follicle-stimulating hormone (FSH) measured twice in patient blood at least one month apart as per the European Society of Human Reproduction (ESHR) guidelines [4–6].

POI is genetically heterogeneous with >100 causative gene variants identified thus far [7, 8]. This number is rising exponentially due to high throughput, cost efficient next generation sequencing methods. Variable genetic cause in POI is partly due to its broad etiology with syndromic, autoimmune, monogenic, and idiopathic causes previously elucidated [9]. Therefore, the identification and classification of causative genes and variants is pertinent to clinical management and prevention of co-morbidities. Co-morbidities of POI include reduced fertility potential, cardiovascular disease, mental health problems, osteoporosis, and premature mortality [1]. Genes identified thus far that contribute to POI include, but are not limited to, genes encoding transcription/translation factors, proteins required for DNA repair/replication, cell differentiation/development, mitochondrial maintenance, hormone signaling, and metabolism [9–11].

Ribonucleic acid (RNA) degradation is critical to the maintenance and stability of cellular life. Stringent parameters for RNA transcript quality, abundance and maturation are regulated by the RNA exosome [12]. The RNA exosome functions as a ubiquitous 3′ - > 5′ exoribonuclease and endoribonuclease, consisting of eleven evolutionary conserved subunits [13]. While initially identified as a central ribosomal processing factor, the role of the RNA exosome was later expanded to include RNA biogenesis and the maturation/degradation of cryptic transcript species in both the nucleoplasm and cytoplasm of eukaryotic cells. More recent literature indicates that the RNA exosome is also necessary for DNA repair by homologous recombination [14].

**Figure 1:**
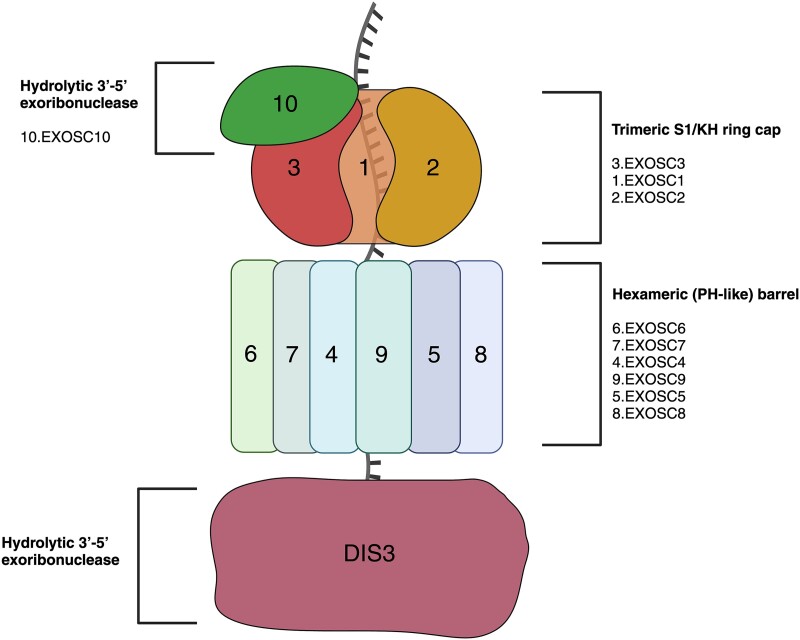
**Structure of the eukaryotic exosome. The eukaryotic exosome consists of nine structural proteins (EXOSC1-9) (indicated 1-9)**. EXOSC1,2,3 form a trimeric cap with a pore opening that allows for ssRNA passage. EXOSC4-9 (indicated 4-9) establish a catalytically inert hexamer barrel ring that facilitates the unidirectional threading of ssRNA. At the base of the hexamer barrel and exit pore is DIS3, a ribonuclease with both exoribonuclease and endoribonuclease activity. Adjacent to the trimeric cap is EXOSC10, (indicated 10) an exoribonuclease that modulates the RNA entry pore. EXOSC10 also facilitates the degradation and exonuclease trimming of entering RNA species. Created with BioRender.com

The general architecture of the RNA exosome is conserved between bacterial, archaeal, and eukaryotic phylogenies [15]. Extensive phylogenetic conservation of the RNA exosome is indicative of the critical role the complex has in cellular life. In eukaryotes the RNA exosome is comprised of a nine- subunit core (EXOSC1-9). Six subunits (EXOSC4-9) establish a catalytically inactive barrel arranged in a hexameric (PH-like) ring wide enough (8–10 Å) to accommodate a single stranded RNA [13] (Figure 1). Due to the width of the hexamer, the barrel arrangement does not accommodate a double stranded RNA [16]. RNA substrates are unwound to single stranded RNAs (ssRNAs) before being threaded unidirectionally through the core barrel [16]. Unlike the archaeal exosome, the eukaryotic exosome core is catalytically inert, suggesting that the role of the exosome core may function as a scaffold structure that requires the association of additional co-factors, enzymes, and nucleases to aid in RNA processing [16]. The remaining three core proteins (EXOSC1, EXOSC2, EXOSC3) form a trimeric S1/KH ring cap. The core exosome proteins (EXOSC1-9) interact with two hydrolytic 3′-5′ exoribonucleases, exosome component 10 (EXOSC10) (rRNA-processing protein 6, Rrp6 in budding yeast) and DIS3 (defective in sister chromatid joining, rRNA-processing protein 44, Rrp44) that both provide enzymatic activity required to cleave RNA [17–19]. Basal DIS3 is located at the base exit pore of the exosome hexameric barrel and functions as both a hydrolytic 3′-5′ exoribonucleases and endoribonuclease [17]. While some eukaryotes, including yeast and *Drosophila* have one Dis3 that is present in both the cytoplasmic and nuclear exosome, humans have two paralogs with DIS3 in the nuclear RNA exosome and DIS3L in the cytoplasmic RNA exosome. DIS3 is an essential effector of RNA degradation, a process that is considered critical to posttranscriptional regulation and cellular function. DIS3 is in the RNase II/R protein family and is considered the predominant ribonuclease subunit responsible for the degradation of promoter upstream transcripts (PROMPTs) and enhancer RNAs (eRNAs) [20, 21].

Aberrant function of the RNA exosome and associated co-factors have been linked to human disease [13]. Variants in genes encoding both structural and catalytic components of the RNA exosome cause diverse pathogenic phenotypes. However, current understanding of how and why RNA exosome derived conditions are isolated to specific tissue types remains rudimentary [22]. Currently, of the eleven identified exosome subunits six have been associated with human disease [23–29]. Basally located DIS3 has been implicated in human diseases associated with aberrant mitotic control [30]. Additionally, patients with Multiple myeloma harboring pathogenic *DIS3* variants were found to have reduced median survival [31, 32].

A dysregulated transcriptome has the potential to disrupt oogenesis and embryonic development post-fertilization [33]. An absence of active transcription in maturing oocytes means that the developing oocyte relies on the active degradation of RNAs to remodel the transcriptome [33]. For degradation to occur the inert six-protein barrel of the RNA exosome must be associated with the enzymatically active components EXOSC10 and DIS3. Therefore, variants impacting the function of DIS3 can have a significant impact on the RNA exosome and can disrupt transcriptome balance.

*Dis3* mouse studies have indicated that DIS3 depletion significantly impacts the fertility of female mice [34]. Oocyte loss of *Dis3* induces premature meiotic arrest of maturing oocytes, whereas global knockout (KO) leads to embryonic lethality [34]. *Dis3* was found to significantly impact RNA degradation in oocytes and its disruption led to insufficient transcription termination paired with the accumulation of intergenic RNA species, ultimately responsible for premature meiotic arrest in DIS3-depleted oocytes [34]. Similar observations have been made in *Drosophila* whereby germline knockdown (KD) of *DIS3* ortholog *Dis3* results in complete infertility in female flies and significant atrophy of ovarian structures with no observable oocytes [35]. Similar cross species observations and significant genetic conservation alludes to a functionally conserved role of DIS3 in maintaining fertility potential.

Here we report a rare homozygous missense variant of *DIS3* in a patient diagnosed with POI. The patient presented at 22 years with secondary amenorrhea, elevated FSH and atrophic ovaries. The variant identified affected a highly conserved residue of the exonucleolytic RNB domain [32, 36]. Given *DIS3* is not a well-established human POI gene with only two compound heterozygous variants previously reported and not functionally characterized, we aimed to model the impact of the patient variant in yeast and *Drosophila.* Our data support a conserved role in fertility between human and *Drosophila* Dis3 and demonstrate that the human DIS3 variant identified in a POI patient has partially reduced function.

This is the second reported case of human *DIS3* variants associated with POI, and the first study to functionally validate variant consequence. This work establishes *DIS3* missense variants as a likely new autosomal recessive cause of POI.

## Materials and methods

### Patient clinical information

The patient presented with secondary amenorrhea at 22 years. Ultrasound revealed she had atrophic ovaries. Blood work taken on two separate occasions reported an elevated FSH of 58 mIU/ml (reference <25 mIU/ml, ESHR guidelines, https://www.eshre.eu/Guidelines-and-Legal/Guidelines/Management-of-premature-ovarian-insufficiency.aspx). Microarray analysis revealed a 46XX karyotype with no significant copy number variation. The patient is from a consanguineous Iranian family. No relatives were known to share the condition of the patient. DNA for other family members was not available. Patient clinical information is consistent with a diagnosis of non-syndromic POI. Information is summarized in Table 1.

**Table 1:** Summary of patients’ information

	**Consanguineous**	**Karyotype**	**Age of dx**	**Menstrual status**	**Hormones**	**Imaging**	**Osteoporotic**
**Patient**	Yes	XX	22	Secondary amenorrhea	FSH 58 mIU/ml	Atrophic ovaries	UN

UN: Unknown

### Whole exome sequencing

Whole exome sequencing (WES) was undertaken on patient blood DNA samples at Victorian Clinical Genetics Services (VCGS). Exome capture with Agilent SureSelect Human All Exon V6 and sequencing on an Illumina NovaSeq 6000 were performed at an average read depth of 80x. WES data were processed using Cpipe [37] and analysed using SeqR (https://seqr.broadinstitute.org/) We used three different approaches to analyse WES data as done previously by [38]. The first phase of analysis was a gene centric approach that focused on the detection of variants in already identified POI genes. For the POI gene-centric approach, high quality rare variants (minor allele frequency [MAF] <0.005) with moderate-high priority (including missense, splicing, predicted loss-of-function) were further considered. The second phase focused on the analysis of any gene with variants fitting a potential recessive inheritance pattern. Similarly, the MAF filter was set at <0.005 and high-quality variants of moderate-high priority were further considered. The third phase involved an analysis of predicted loss of function (LoF) variants in any gene. This phase was not restricted to bi-allelic variants so a more stringent MAF of <0.0001 was used, as deleterious dominant variants are less likely to exist in a healthy population.

### *S. cerevisiae* model

The analysis of yeast mutants was performed as previously described [39, 40]. Briefly, a strain that had the chromosomal copy of *DIS3* (also known as *RRP44*) deleted and that expressed the essential *DIS3* from a *URA3* plasmid (yAv1121; *Matα, ura3-∆0, leu2-∆0, his3-∆1, dis3∆::HYG [DIS#,URA3]*) was transformed with one of four *LEU2* plasmids: *Wild-type DIS3* (pAv344) [39], *dis3-H841Y* (pAv1569), *dis3-H841A* (pAv1568), or empty vector (pRS415) [41]. Transformants were selected on SC-Ura-Leu, serially diluted and plated either on 5FOA (5-fluoro-orotic acid) containing media (selecting for cells that had lost the *URA3* plasmid) or on SC-Leu media (as control). The ability to grow on 5FOA-containing media reflects that the *URA3* plasmid can be lost, and thus that the *dis3* allele on the *LEU2* plasmid is functional. The point mutations in pAv1568 and pAv1569 were generated by the QuikChange method and verified by sequencing the whole plasmid (Plasmidsaurus).

### *Drosophila melanogaster Dis3* KD model

RNA interference (RNAi) line for *Dis3* was obtained from the Bloomington *Drosophila* Stock Centre (BDSC). The *Drosophila* line utilized was *Dis3* RNAi (BL67919). Additional *Drosophila* lines included control strain *w^1118^* (BL5905), *nanos*GAL4 (*nos*) driver generated from UAS-GAL4; GAL4::VP16-*nos*.UTR (BL4937 and BL5938) all from BDSC. The *Traffic-jam* GAL4 (*Tj*) driver used (DGRC104055) was obtained from the Kyoto Stock Centre. All long-term stock storage was maintained at 25°C on a molasses-based medium.

To establish KD of *Dis3,* the RNAi transgenic line was crossed to both *Tj-GAL4* and *nanosGAL4. Tj-GAL4* drives the KD of *Dis3* in germline associated somatic cells during ovarian development and oogenesis [42, 43]. In turn, *nanosGAL4* drives the KD of *Dis3* in germ cells from the apical germarium until the mature oocyte [44, 45]. Controls used in this work were *w1118* flies crossed with the respective driver (*Tj* or *nanos*). *Drosophila* crosses of RNAi lines to GAL4 drivers were raised on molasses-based medium supplemented with dried yeast. When crossed to *Tj* and *nos* drivers, *Drosophila* were raised at 18°C for initial cross and until larvae enclosure, and then moved to 29°C until pupal enclosure. The cross was then maintained at 29°C for 3–5 days to allow for the F1 generation to mate prior to ovary dissection, immunostaining, and fluorescent microscopy analysis. >15 ovaries were observed from >2 technical replicate crosses. Ovaries were dissected from the progeny and were immunostained using anti-Fasciclin 3 (FasIII) to visualize somatic stem cell derived follicular cells and polar cells, anti-*vasa* that marks the VAS protein that localizes to nucleoplasm of ovarian germ cells, and nuclear stain 4′,6-diamidino-2-phenylindole (DAPI) [46–48]. Immunostaining was done as per [49].

### Generation of human DIS3 transgenic lines

DNA sequences of the open reading frame transcript corresponding to wild-type human *DIS3* and human *DIS*${3}^{H774Y}$ were synthesized by Azenta/Genewiz and directionally cloned into pUASTattBusing XhoI and XbaI restriction sites that were added to the 5′ and 3′ ends of the sequences, respectively. Transgenic flies were generated by BestGene, Inc. Both transgenes were inserted into the identical genomic location on chromosome 3 (attP2) using phi C31 integrase [50].

### Statistical methods

Calculations for significance used either a two-proportion z-test (Figure 6) or an unpaired t-test (Figures 7, 8) with p-values for significance set at *P* < 0.05. Statistical analysis was undertaken in Prism10 software.

## Results

### Identification of DIS3 homozygous missense variants

Gene-centric and variant-centric analysis was performed on the patient. 29 rare moderate-high priority variants were identified in known or candidate POI genes. Twenty-four of the 29 POI-related variants were heterozygous and did not fit with the known mode of POI inheritance associated with the gene and/or had low pathogenicity prediction based on online algorithms and conservation scores. One gene, *MTF1* had two variants of interest suggesting possible compound heterozygosity. *MTF1* was not pursued for follow up due to the low pathogenicity prediction of variants in-*silico* (Supplementary Figure S1).

Homozygous missense variants were observed in three POI candidate genes; *DIS3, RAD51B* and *APC2*. *RAD51B* is a strong POI candidate gene due to its role in homologous recombination. However, this gene was not considered the strongest candidate due to the low in silico pathogenicity predictions and poorly conserved altered residue (Supplementary Table S1).

*APC2* has been considered a POI-candidate gene because APC2 deficiency in mice leads to disrupted ovarian WNT signaling and female subfertility [52]. Due to benign PolyPhen *in-silico* pathogenicity prediction, *APC2* was not considered the strongest candidate (Supplementary Table S1).

In the second phase of analysis, focusing on genes with variants that could fit recessive inheritance, variants in *DIS3, MTF1, RAD51B* and *APC2* were again identified along with variants in *MGA. MAX Dimerization Protein (MGA)* acts as a tumor suppressor gene [53]. Although *in silico* predictions indicated this was a potentially deleterious variant, no direct link with infertility had been reported in association with this gene.

**Figure 2:**
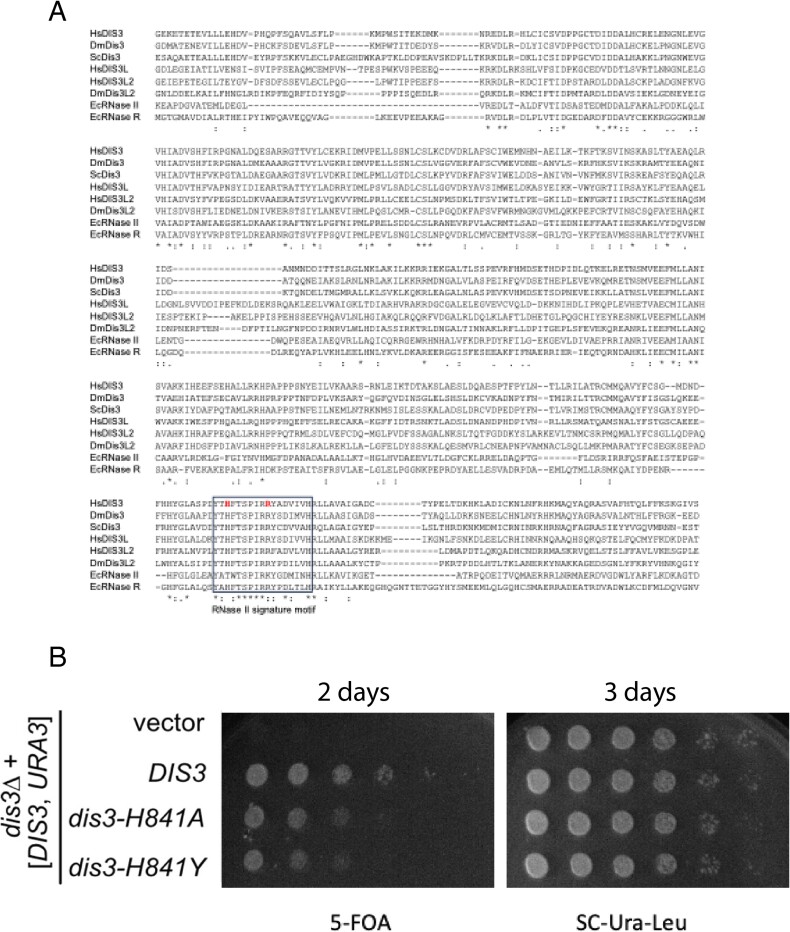
**DIS3 is well conserved across species but in *Saccharomyces cerevisiae DIS3* variant p.His841Tyr impairs mitotic growth**. A. Shown is an alignment of the catalytic domains of human DIS3 (residues 424-847) and the homologs discussed in the text. HsDIS3: *Homo sapiens* DIS3; DmDis3: *Drosophila melanogaster* Dis3; ScDis3: *S. cerevisiae* Dis3; HsDIS3L: *H. sapiens* DIS3L; HsDIS3L2: *H. sapiens* DIS3L2; EcRNase II: *Escherichia coli* RNase II and EcRNase R: *E. coli* RNase R. The box highlights the RNase II signature motif. The residues mutated in our POI patient (His774), and Johnstone et al previous POI patient (Arg781) are highlighted [35]. His774 of human DIS3 aligns with His841 of the yeast orthologue Dis. B. Changing His841 in yeast to either Tyr (as observed in our POI patient) or Ala impairs mitotic growth at 25${}^{{}{\circ}}\mathrm{C}$. This analysis was repeated with five biological replicates all of which yielded the same result. Yeast was grown for 2 and 3 days at 25${}^{{}{\circ}}\mathrm{C}$

Therefore, after filtering and in-*silico* analysis we considered the homozygous missense variant observed in *DIS3* as the top candidate. The patient has a homozygous missense variant c.2320 C > T that results in a predicted amino acid change from histidine to tyrosine in *DIS3* at position 774, p.His774Tyr. The variant is in a highly conserved residue, has high pathogenicity in-*silico* predictions as well as an association in mice with a significant infertility phenotype [34] (Supplementary Table S1). His774 is part of the RNase II signature motif that defines the family of RNase II-like enzymes (Figure 2 A). This histidine is highly conserved in eukaryotic Dis3 and Dis3L enzymes, and even in *Escherichia coli* (*E.coli*) RNase R, but not in the namesake member of the family RNase II (where the corresponding residue is Thr494).

At the time of analysis, no other *DIS3* variant had been described in a human POI patient. However, more recently, an Arg781Thr variant has been described in another human POI patient. Strikingly, this is also a highly conserved residue in the RNase II signature motif, and His774 and Arg781 are adjacent in the 3D structure of DIS3, contacting the same phosphate of the RNA substrate and possibly mediating translocation of the substrate through the active site (see discussion) strengthening our assertion that this gene and variant are causative [35, 54].

### The DIS3 patient variant p.His774Tyr impairs mitotic growth in *S. cerevisiae*

*DIS3* variants often confer mitotic defects that result in premature cell cycle arrest, unfavorable centrosome amplification and defects in spindle assemble checkpoints resulting in pathogenic aneuploidy and genomic instability [55]. In *S. cerevisiae* (yeast) *DIS3* is an essential gene, and some point mutants have been noted to confer mitotic spindle assembly defects that are believed to impact mitotic fidelity [56, 57]. To determine whether the human *DIS3* variant is deleterious, we modelled it in yeast, which has the most extensively characterized RNA exosome. Sequence alignment identified yeast His841 as the residue equivalent to His774 in human DIS3 (Figure 2 A). We therefore mutated His841 to tyrosine to mimic the patient variant. In addition, we also generated a His841 to Ala mutation. We reasoned that the polar interaction of His84 with the RNA backbone [18] may be maintained by tyrosine and that mutating His841 to Ala might better reveal the function of this residue. We used a *dis3* KO yeast strain complemented with a wild-type *DIS3* gene on a *URA3* plasmid. This strain is unable to grow on media containing 5-Fluoro-orotic acid (5-FOA) because 5FOA selects for cells that have lost the *URA3* plasmid, and consequently *DIS3*. This strain was transformed with either a wild-type *DIS3* plasmid, a *dis3-H841Y* mutant, a *dis3-H841A* mutant or an empty vector. Transformants were then serially diluted and spotted on either 5FOA-containing media, or 5FOA-free media. As expected, the strain transformed with empty vector was unable to grow on 5-FOA media (Figure 2 B). Further, the transformants expressing either *DIS3*, *dis3-H841Y* or *dis3-H841A* were able to grow on the selective 5-FOA media, however growth is impaired for the mutant plasmids (Figure 2 B). This indicated that in yeast, the p.His841Tyr does not eliminate, but rather impairs mitotic growth. We and others have previously shown that mutations that eliminate catalytic activity slow growth [39, 58, 59]. Thus, it can be concluded that *dis3-H841Y* does not completely eliminate catalytic activity, consistent with previous biochemical characterization of the RNase II family (see discussion) [39, 58, 59].

## Knockdown of *Dis3* in *drosophila* procures a dysgenic ovarian phenotype

### *Drosophila Dis3* is the ortholog of human *DIS3*

Given that in mice oocyte specific *Dis3* KO produces a sub-fertile phenotype and meiotic failure, and observations that our patient variant impairs mitotic growth in yeast we decided to further model the patient variant in *Drosophila*. The orthologue of human *DIS3* in *Drosophila, Dis3,* encodes a protein with 54% identity and 70% similarity to the human DIS3 protein (Supplementary Figure S1). The affected residue at human position 774 correlating to *Drosophila* position 783 (Supplementary Figure S1). Information taken from (https://flybase.org/). We therefore pursued a model of our patient variant in *Drosophila* using a *Dis3* KD rescue approach. However, prior to attempting a rescue experiment with the identified patient variant we wanted to determine the impact endogenous *Drosophila Dis3* depletion had on ovarian development. This was assayed in both a germline and somatic cell context.

### *Dis3* KD induces an “ovary-less” phenotype in *drosophila* when driven in somatic gonadal precursor cells

**Figure 3:**
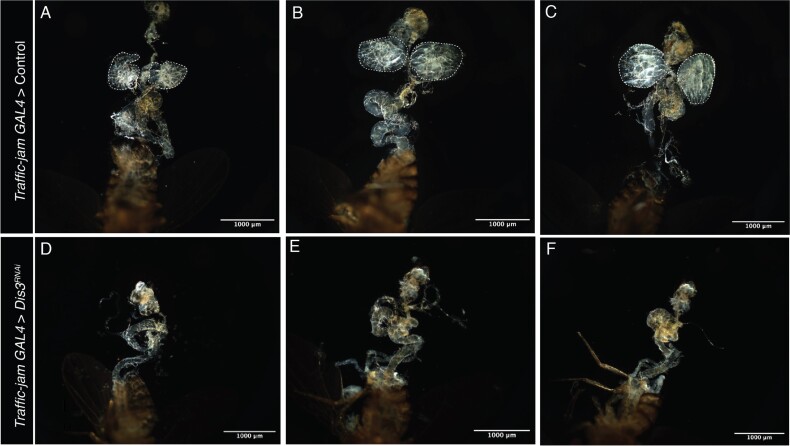
***Dis3* KD via *Traffic-jam* GAL4 in somatic gonadal precursor cells of the *Drosophila* ovary induces an “ovary-less” phenotype**. Control (W1118) *Drosophila melanogaster* crossed with *Traffic-jam* GAL4 somatic driver (upper panels) showing three biological replicates with dotted line indicating *Drosophila* ovarian structures (A,B,C). *Dis3* RNAi (BL67919) crossed with *Traffic-jam* GAL4 somatic driver (lower panels) to induce KD, indicating a complete absence of ovarian structures in three biological replicates (D,E,F). All flies were reared at 18${}^{{}{\circ}}\mathrm{C}$ until larval eclosure then switched to 29${}^{{}{\circ}}\mathrm{C}$ until pupation and adult eclosure. Adults were mated for 3-5 days at 29${}^{{}{\circ}}\mathrm{C}$ and then dissected. Scale bar = 1000$\mu m,\mathrm{n}=20\ \mathrm{flies}$

To determine the impact of *Dis3* depletion on ovarian development, we generated *Drosophila* with *Dis3* RNAi driven under the Traffic-jam (*Tj*) promotor (*Tj*> $Dis{3}^{RNAi}$*)*. Tj is expressed in somatic cells that are directly associated with germline cells of the *Drosophila* ovary, such as intermingled cells (ICs), cap cells, escort cells and follicle cells, throughout ovarian development and oogenesis [42, 43]. Dissection of F1 *Tj*> $Dis{3}^{RNAi}$ progeny revealed no visible ovarian structures (Figure 3) or germ cells, indicating ovarian development is severely disrupted upon the depletion of *Dis3*. (Figure 3).

### *Dis3* KD in *drosophila* germline produces atypical ovarian development

**Figure 4:**
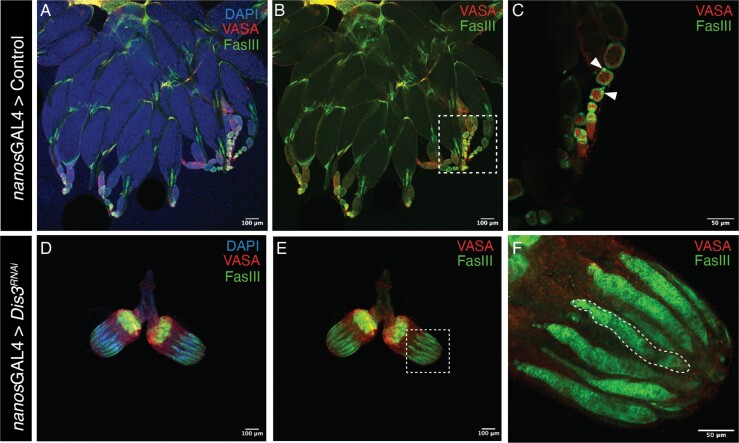
**Depletion of *Dis3* in the *Drosophila* germline causes aberrant ovarian development**. When *Dis3* is depleted in the *Drosophila* germline via *nanos*GAL4 (*nanos* > *Dis*${3}^{RNAi}$) an ovarian structure with no observable oocytes, germ-cells and rudimentary ovarioles is observed (D,E,F). This is visualised by the absence of germline specific VAS protein that encompasses the nuclei of germline stem cells as indicated in the control by arrowheads in C. Somatic staining of follicle somatic hub, polar and terminal epithelial cells (FasIII) is also disrupted and punctate throughout the rudimentary ovariole structure (F, dotted line). Representative confocal images of control ovary (A,B,C) with observable germ cells (C, arrowhead)(n = 22 ovaries) and *nanos* > *Dis*${3}^{RNAi}$ (D,E,F)(n = 20 ovaries).

To determine the impact of *Dis3* depletion in germ cells, we generated a *Drosophila* line with *Dis3* RNAi under the control of germline driver *nanos*GAL4 (*nanos* > *Dis*${3}^{RNAi}$). *nanos* drives the KD of *Dis3* from the apical germarium until the mature oocyte. Dissection of F1 progeny revealed the presence of ovarian structures but an absence of ovariole (chain of developing egg chambers), germarium (structure that contains undifferentiated germ cells) and germ cells (Figure 4). When compared to the *nanos*GAL4 > Wild-Type (WT) ovaries (Figure 4, A-C) *nanos*-driven *Dis3* KD leads to small, poorly formed ovaries, akin to those of a rudimentary larval ovary (n = 20 ovaries) (Figure 4, D-F) [42, 60]. The absence of perinuclear VASA in immunostaining, indicates the absence of germ cells in the ovary upon depletion of *Dis3* in the germline (Figure 4, F). Staining of somatic follicle hub and terminal epithelial cells via FasIII protein highlights the developing ovariole epithelia as well as polar cells at both the anterior and posterior of the developing egg chambers in WT Figure 4, A-C. The differentiation of pre-polar cells into polar cells and stalk cells maintains egg chamber separation which is essential to developing *Drosophila* ovarioles [61, 62]. Upon *Dis3* depletion FasIII staining indicates the presence of rudimentary ovarioles (Figure 4, D-F). However, FasIII staining is disorganized and not localized to the follicular epithelial or polar cells and there is a complete absence of identifiable egg chambers or distinct germarium structures (Figure 4 E, F). In sum, depletion of *Dis3* in the *Drosophila* ovarian germline causes dysgenic development.

## The human DIS3 variant p.His774Tyr (hDIS ${\mathbf{3}}^{\mathbf{p.H774Y}}$) has reduced function in ovarian development

### Somatic human *DIS3* expression substantially rescues ovarian function in somatic *DIS3* KD *drosophila*

To determine if the *DIS3* c.2320C > T /p.His774Tyr variant is causative of POI in our patient we modelled the patient variant in *Drosophila* ovaries. To achieve this, we generated two *Drosophila* lines, one containing the human WT *DIS3* (*hDIS3*) and one expressing human *DIS3* with the patient variant, *p.His774Tyr (*hDIS${3}^{\mathrm{p}.\mathrm{H}774\mathrm{Y}}$*)*(see methods) under the UAS-promoter system. These flies were genetically recombined with BL67919 *Drosophila* harboring *Dis3* RNAi to deplete endogenous *Drosophila Dis3* (*dDis3*). Both lines were then crossed to somatic driver *Traffic-jam* GAL4 (*Tj*) to perform a rescue experiment.

**Figure 5:**
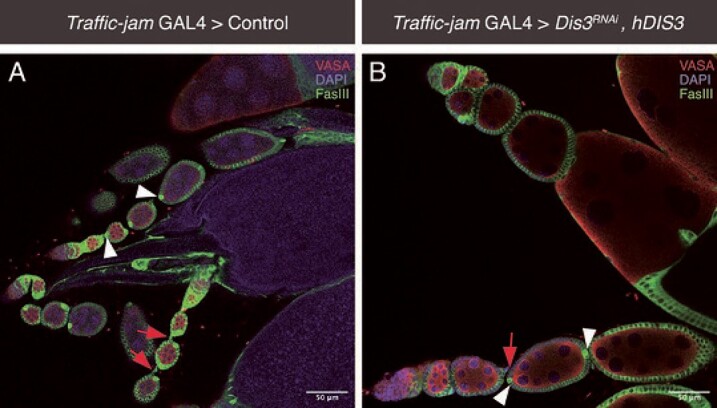
**Human *DIS3 (hDIS***  $\mathbf{3}$) **substantially rescues *Drosophila* ovarian development in the absence of endogenous *Dis3 (dDis3)****.* A. *Drosophila* expressing endogenous *Dis3* (*dDis3)* produce ovariole structures that develop linearly. Polar cells are organized both anteriorly and posteriorly in each developing egg chamber (A, arrowheads). Each egg chamber is separated via interfollicular stalk cells that aid in coordinated oocyte development (A, arrows). B. When *Traffic-jam* GAL4 (*Tj*) drives endogenous *dDis3* depletion and the expression of *hDIS3* (*Tj>*$Dis{3}^{RNAi}, hDIS3$) *hDIS3* substantially rescues *Drosophila* ovarian development and function. The majority of ovarioles in *Tj>*$Dis{3}^{RNAi}, hDIS3$ had normal ovariole structure (lower ovariole). *Tj>*$Dis{3}^{RNAi}, hDIS3$ WT ovarioles have both polar cells (B, white arrowheads) and interfollicular stalk cells (B, red arrowheads) located similarly to that of the control (A). Scale bar = 50$\mu m$, *Tj > Control* n = 22 ovaries, $Tj> Dis{3}^{RNAi}\ hDIS3$*n = 15 ovaries*

When BL67919 *Drosophila Dis3* RNAi and *hDIS3* were both driven under the *Tj* promotor ($Tj> Dis{3}^{RNAi}, hDIS3$*)* hDIS3 was able to substantially rescue the “ovary-less” phenotype observed in $Tj> Dis{3}^{RNAi}$ (Figure 5). Ovarian structures were clearly visible with linear developing ovariole structures as well as normally oriented follicle and polar cells. Developing egg chambers were separated by a discernible series of stalk cells like that of the control (Figure 5 B, red arrowheads). These rescue data demonstrate that human *DIS3* has a degree of functional redundancy in *Drosophila* and confirms that the ovarian phenotype observed in KD flies is indeed attributable to *Drosophila* Dis3 depletion and not off-target effects. Whilst rescue of endogenous *Dis3* by *hDIS3* is observed, there were some subtle changes when compared to the *Tj* > Control suggesting incomplete complementation. These are discussed in comparison with hDIS${3}^{\mathrm{p}.\mathrm{H}774\mathrm{Y}}$ below.

### h $\mathbf{DIS}{\mathbf{3}}^{\mathbf{H774Y}}$ exhibits reduced ability to rescue ovarian loss induced by downregulation of *drosophila Dis3*

Similar to rescue by *hDIS3,* gross ovarian morphology was restored by expression of hDIS3^p.H774Y^ in the *Drosophila* ovary. This suggests that the human variant does not abolish DIS3 function. To investigate whether the p.H774Y variant may be hypomorphic, we assayed for more subtle changes in ovarian morphology, such as aberrant ovariole development.

**Figure 6:**
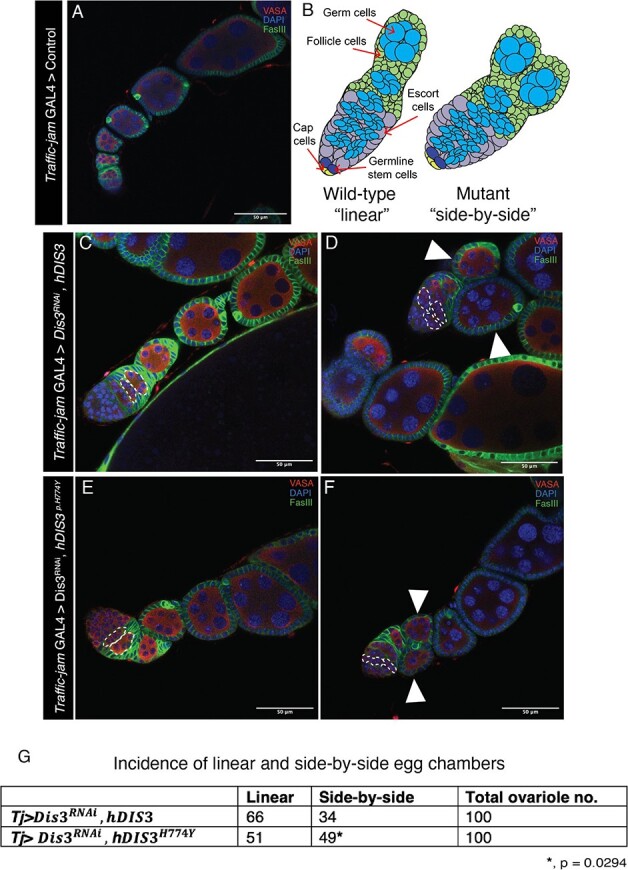
**Human *DIS3* variant *p.H774Y* leads to increased ovariole dysmorphology**. (A) Typical linear arrangement of ovarioles as seen in *Traffic-jam* GAL4 > Control. (B) Schematic of Wild-type “Linear” germarium and S2-S3 egg chambers and mutant “side-by-side”. Developing germline cysts are encapsulated individually by follicle cells with single cyst budding in WT. In the mutant, dual cysts with follicle cell bi-layers and disordered cysts packaging in germarium can be seen (right). Cap cells, germline stem cells, and escort cells are indicated. Adapted from [51]. Linear arrangement of developing cysts is observed in both to *Tj>*$Dis{3}^{RNAi}, hDIS3$ (C) and *Tj*> $Dis{3}^{RNAi},$*hDIS*${3}^{H774Y}$ (E). *Tj>*$Dis{3}^{RNAi}, hDIS3\kern0.5em$ and *Tj*> $Dis{3}^{RNAi},$*hDIS*${3}^{H774Y}$ both present with a “side-by-side” arrangement of developing S2-S3 egg chambers in *Drosophila* ovarioles (D,F, arrowheads). “Side-by-side” egg chambers have been previously observed and linked to germline cyst packaging defects in *Drosophila* germarium whereby cysts are located adjunct instead of sequentially during development. Sequential cyst formation can be seen in C and E dotted line whilst adjacent cysts can be seen in D and F, dotted line. Frequency of “side-by-side” egg chambers for both *Tj>*$Dis{3}^{RNAi}, hDIS3$ and *Tj*> $Dis{3}^{RNAi},$*hDIS*${3}^{H774Y}$ were calculated from n = 100 ovarioles of each genotype (G). Statistical significance between the two groups was calculated using a two-proportion z-test with confidence set at 0.05. There was a greater proportion of “side-by-side” ovarioles in *Tj*> $Dis{3}^{RNAi},$*hDIS*${3}^{H774Y}$ 49% when compared to *Tj>*$Dis{3}^{RNAi}, hDIS3$ 34% (^*^, P = *0.0294, n = 100 ovarioles)* Scale bar = 50$\mu m$

In *Tj>*$Dis{3}^{RNAi}, hDIS3$ whilst gross morphology was predominantly rescued (Figure 6 C), there were subtle changes suggesting rescue was incomplete as occasional ovarioles within *Tj>*$Dis{3}^{RNAi}, hDIS3$ rescue ovaries were dysmorphic. Specifically, in the packaging and development of budding egg chambers whereby a side-by-side arrangement was observed 49% of the time in 100 ovarioles assayed Figure 6 D, G. *Tj>*$Dis{3}^{RNAi}, hDIS3$ ovarioles had absent or inconsistently observed/positioned polar cells. *Tj>*$Dis{3}^{RNAi}, hDIS3$ ovarioles with dysgenic polar cell arrangement also exhibited a distinct “side-by-side” development of egg chambers at stages 2–3 (S2-S3) instead of the WT linear arrangement (represented in Figure 6 A–B) (Figure 6 D, white arrowhead). Aberrant “side-by-side” arrangement of egg chambers has been previously observed and linked to varying packaging defects in the process of germline cyst encapsulation by follicle cells in the germarium [63–70]. However, fewer cases have been documented whereby paired cysts in the germarium become individually encapsulated by a follicular epithelium. Bi-layered follicular epithelium at the region of fusion of “side-by-side” egg chambers has been previously noted in *Pak* mutants [51]. This observation is similarly observed in *Tj>*$Dis{3}^{RNAi}, hDIS3\kern0.5em$ as a defined bilayer of follicular epithelial cells can be observed between the “side-by-side” paired egg chambers (Figure 6 D). Despite “side-by-side” events occurring 34% of the times in 100 ovarioles assayed, rescue of *Dis3* depletion with *hDIS3* was evident with this “side-by-side” phenotype significantly exacerbated (*P* = 0.0294) in the presence of the H774Y variant (Figure 6). Figure 3 and Figure 5 reflect a significant conservation between human *DIS3* and *Drosophila Dis3*.

Akin to *Tj>*$Dis{3}^{RNAi}, hDIS3\kern0.5em$ a “side-by-side” arrangement of early S2-S3 egg chambers is similarly observed in *Tj*> $Dis{3}^{RNAi},$ hDIS${3}^{H774Y}$ (Figure 6 F, white arrowhead). In *Tj*> $Dis{3}^{RNAi},$ hDIS${3}^{H774Y}$ a defined bilayer of follicular epithelial cells can be observed between the “side-by-side” paired egg chambers similar to *Tj>*$Dis{3}^{RNAi}, hDIS3\kern0.5em$(Figure 6 D and F, white arrowheads). Upon closer examination, this packaging defect appears to originate in the germarium of both genotypes (Figure 6 D and F, dotted line). Here germ cells fail to aggregate into singular disc shaped cysts as seen in Figure 6 C and E, dotted line. Instead, the cysts are aberrantly paired in a germarium that is disordered and structurally shorter and wider than the *Tj* > Control (Figure 6 A–F).

Finally, both *hDIS3* and h*DIS*${3}^{\mathrm{p}.\mathrm{H}774\mathrm{Y}}$ demonstrate alternative singular and paired germarium cysts and paired/unpaired S2-S3 egg chambers within the same ovary (Figure 6, C-F). This variation in phenotype within a single ovary has been noted previously in literature [51]. Analysis of 100 ovarioles for both *hDIS3* and h*DIS*${3}^{\mathrm{p}.\mathrm{H}774\mathrm{Y}}$ revealed a significant (^*^, *P* = 0.0294) increase in paired egg chambers in *Tj*> $Dis{3}^{RNAi},$*hDIS*${3}^{H774Y}$ when compared to *Tj>*$Dis{3}^{RNAi}, hDIS3\kern0.5em$ (Figure 6 G). This indicates that hDIS${3}^{\mathrm{H}774\mathrm{Y}}$ likely does not function to the same extent as *hDIS3*, supporting the notion that the probability c.2320C > T variant is a hypomorphic allele.

### Rescue with hDIS ${\mathbf{3}}^{\mathbf{p.H774Y}}$ induces premature egg chamber degradation in the *drosophila* ovary

**Figure 7:**
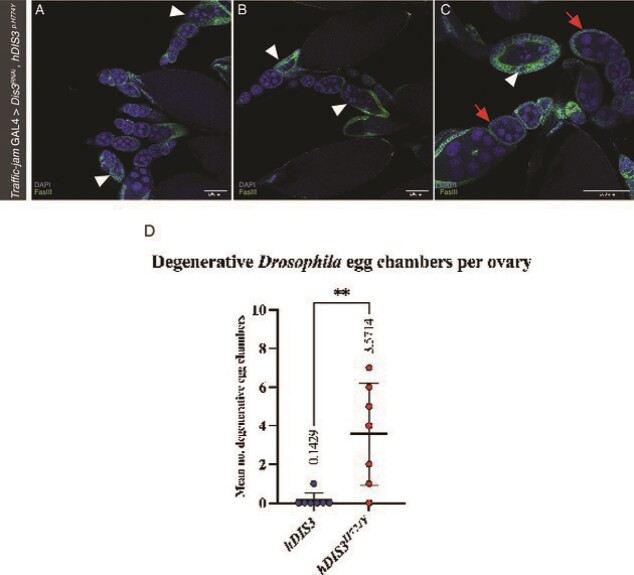
**p.*H774Y* induces increased egg chamber degradation in the *Drosophila* ovary**. Death of stages 7–8 egg chambers is observed upon *Tj*> $Dis{3}^{RNAi},$*hDIS*${3}^{H774Y}$. This is observed via irregular, lobed presentation of DAPI-stained nurse cell nuclei in A–C. Aberrant DAPI staining of nurse cell nuclei is evidence of DNA fragmentation and chromatin condensation and is the first stage in *Drosophila* egg chamber degradation. Upon nurse cell nuclear condensation surrounding follicle cells become phagocytotic and the lineage expands to engulf nurse cell debris. This can be seen via an increase of FasIII staining in C. Apoptotic egg chambers are indicated via arrowheads and healthy/normal/control egg chambers are indicated via arrowheads. Mean count of apoptotic *Drosophila* egg chambers D. Scale bar = 100 $\mu m$, ^**^, P = 0.0052, error bars indicate standard deviation, n = 14 ovaries

*Tj*> $Dis{3}^{RNAi},$ hDIS${3}^{H774Y}$ ovaries demonstrate increased egg chamber degeneration when compared to *Tj>*$Dis{3}^{RNAi}, hDIS3$ rescue flies. There are two primary checkpoints in *Drosophila* egg chamber development [71, 72]. The first is in region two where germline cysts are surrounded by follicle cells in the germarium to prepare for cyst budding. The second is in developmental stages 7-8 which is widely considered as a mid-oogenesis checkpoint [71, 73]. The first indication of mid-oogenesis degeneration in the germline is identifiable via irregular morphology of Nurse Cell (NC) nuclei [74]. Irregular NC nuclei were observed in *Tj*> $Dis{3}^{RNAi},$ hDIS${3}^{H774Y}$*Drosophila* by the globular distribution of DAPI staining indicative of NC DNA fragmentation (Figure 7A-B, arrowheads) [75]. NC death stimulates phagocytotic activity of the surrounding follicle layer [76]. The follicular cells then transitions from a quiescent support state to a phagocytotic state for complete efferocytosis of the NC debris [77]. Once complete the remaining follicular cells undergo auto-apoptosis. Late-stage chromatin condensation is observed in hDIS${3}^{\mathrm{p}.\mathrm{H}774\mathrm{Y}}$ rescue *Drosophila* (Figure 7, arrowheads). Expansion of the follicle layer is also observed in Figure 7C, arrowheads. The checkpoints for egg chambers in stages 7-8 likely reflect developmental changes in the egg chamber whereby sensitivity to aberrant function and genetic aberration is increased prior to energetically taxing vitellogenesis that occurs post stage 8 [77]. Therefore, egg chambers with incorrect follicular cell layer development, germ cell abundance and altered genetic background become targets of programmed cell death [74, 75]. A total of 14 ovaries from seven different *Drosophila* were dissected for both *Tj>*$Dis{3}^{RNAi}, hDIS3\kern0.5em$ and *Tj*> $Dis{3}^{RNAi},$ hDIS${3}^{H774Y}$. None of the egg chambers observed exhibited germline degeneration prior to the mid-oogenesis checkpoint at stage 7-8. A statistically significantly (^**^, *P* = 0.0052) increase in degradation at stage S7-8, however, was noted in *Tj*> $Dis{3}^{RNAi},$ hDIS${3}^{H774Y}$ ovaries with a mean occurrence of 3.570 degradation events per *Drosophila* ovarian pair compared to 0.1429 observed in *Tj>*$Dis{3}^{RNAi}, hDIS3$ flies (Figure 7D). This indicates that the presence of p.H774Y variant increases the occurrence of egg chamber degradation indicating a likely deleterious nature of the p.H774Y variant.

### *Drosophila Dis3* depletion in the germline is rescued similarly by both human DIS3 (*hDIS*  $\mathbf{3}$) and human variant *DIS*  ${\mathbf{3}}^{\mathbf{p.H774Y}}$ (h*DIS*${\mathbf{3}}^{\mathbf{p.H774Y}}$)

**Figure 8:**
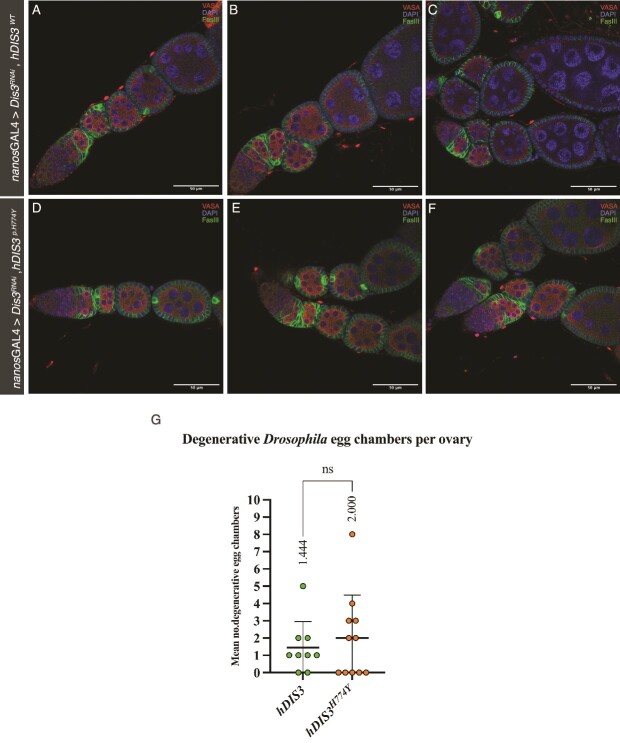
**Similar rescue of *Dis3* depletion in the germline is observed when *hDIS3***  $\mathbf{and}$***hDIS***${\mathbf{3}}^{\boldsymbol{p.H}\mathbf{774}\boldsymbol{Y}}$  **are driven via *nanos*GAL4**. Both *hDIS3*$\mathrm{and}$*hDIS*${3}^{p.H774Y}$ similarly rescue *dDis3* when driven via germline driver *nanos*GAL4. This can be seen in the similar structure and arrangement of ovarioles and egg chamber development shown in A-F. There is no significant increase in S7-8 egg chamber degradation in *hDIS*${3}^{p.H774Y}$ when compared to *hDIS3* (G), ns = 0.5655, error bars indicate standard deviation, *hDIS3* n = 9 ovaries, *hDIS*${3}^{p.H774Y}$ n = 11 ovaries

When *hDIS3*  $\mathrm{and}$*hDIS*${3}^{p.H774Y}$ are driven via *nanos*GAL4, in germline-*Dis3* depleted *Drosophila,* no significant difference in ovarian morphology is observed. In both instances *hDIS3*$\mathrm{and}$*hDIS*${3}^{p.H774Y}$ rescue the phenotype observed in Figure 4. Distinct ovariole and egg chamber development is observed with sequential cyst budding akin to those of the *nanos*GAL4 > Control ovaries Figure 8 A–C. Expression of either *hDIS3 hDIS*${3}^{p.H774Y}$ rescues the lack of egg chamber development and oocytes observed in *nanos*-driven *Dis3* depleted *Drosophila.* These results indicate that, in this context, the variant *hDI*$S{3}^{p.H774Y}$ allows for ovarian development to proceed similarly to *hDIS3.* Further, upon the analysis of *hDIS3*  $\mathrm{and}$*hDIS*${3}^{p.H774Y}$ driven via *nanos* there was no significant difference in degradation of S7–S8 egg chambers (Figure 8).

## Discussion

Our data report the second unrelated case of biallelic *DIS3* variants in a patient presenting with POI. Our patient was diagnosed with POI at 22 years and presented with elevated FSH, atrophic ovaries and secondary amenorrhea (Table 1). A rare homozygous missense variant in *DIS3* was identified as the leading candidate for further analysis. This *DIS3* variant c.2320C > T is predicted to cause an amino acid change from histidine to tyrosine in a highly conserved residue.

Prior to our analysis, no variants in *DIS3* had been reported in association with human POI. Recently, however, compound heterozygous variants have been reported in two sisters with POI, a Arg781Thr missense variant and in-frame deletion of Arg624 [35]. Based on structural data of the human RNA exosome and biochemical characterization of related bacterial enzymes, His774 and Arg781 may dictate the final product of RNA digestion. The related *E. coli* enzymes RNase R and RNase II differ in the final product: RNase R degrades RNA from the 3′ end until the RNA is 2-3 nts long, while RNase II stops when it reaches the final 4-5 nucleotides. Both His774 and Arg781 are conserved in RNase R (His565 and Arg572) but not in RNases II (Thr494 and Lys501; Figure 2). Strikingly, mutating either of these residues in RNase R changes the end product to that of RNase II [78]. Based on these analyses, a possible effect caused by the POI mutations in *DIS3* is that the same substrate RNAs are targeted, but that a longer final product is released. For capped RNAs, degradation of these final products requires DcpS, which is only active on very short RNAs [79]. Thus, we speculate that the final product of DIS3-His774Tyr (and DIS3-Arg781Thr) is a poor substrate for DcpS, leading to accumulation of short-capped mRNA fragments. One explanation for the link to POI is that over time ovaries become sensitive to overproduction of 4-5nts RNA remnants.

Johnstone et al [35] assessed the impact of *DIS3* in ovarian development via the KD of *Dis3* in *Drosophila* via a *Maternal Triple Driver*-GAL4. *Maternal Triple Driver*-GAL4 drives transgene expression in the germarium and throughout oogenesis in germline cells of the ovary [80]. However, this study lacked functional validation of their specific patient variant in their *Drosophila* model.

Functional validation is critical for clinical applicability of research findings. Although *Dis3* is indisputably necessary for proper ovarian development, whether human missense variants disrupt *DIS3* function and lead to POI requires further elucidation. We initially used yeast modelling to assay the impact of our patient variant, which indicated a small defect in mitotic replication. Similar yeast models have been used to model some other exosomopathies. Only single amino acid changes that cause subtle defects on the essential RNA exosome are compatible with a completed pregnancy and birth. These subtle effects sometimes cause effects in a yeast model, but in many cases they do not detectably affect growth [81, 82]. Given *DIS3* has been implicated in both mitosis and meiosis, we considered that this yeast model may not have been optimal and pursued *Drosophila* modelling so we could investigate the *in vivo* impact of the variant on clinically-relevant ovarian development. RNAi depletion in somatic and germline ovarian cells indicates a functional requirement for *Dis3* in both tissue types to facilitate normal ovarian development.

The *Drosophila* equivalent of mouse and human granulosa cells, in an embryological context, are the follicle cells [83]. The somatic cell driver *Traffic-jam* GAL4 drives the KD of *Dis3* RNAi in somatic follicle cells of the *Drosophila* ovary. Given the striking “*ovaryless*” phenotype observed when *Dis3* is knocked down via *Traffic-jam* GAL4 it is reasonable to speculate that Dis3 has a critical role in granulosa cell development.

Previous work has highlighted an important relationship between the *Drosophila* oocyte and its support cells, nurse and follicle, during development. When *Drosophila* oocyte specification is lost, nurse cells and entire egg chambers undergo apoptosis [83]. This finding is similarly observed in our data with an increased degradation of *Drosophila* egg chambers in the presence of the human *DIS3* variant in a somatic cell context. This also alludes to a critical role of both the follicle and nurse cell populations in supporting oocytes development in *Drosophila.* This relationship suggests that the role of *DIS3* in oocyte maturation and development, in both a somatic and germline context, may be similar in higher eukaryotes such as mice and humans given the conserved role of these cell types. *hDIS3^H774Y^* had a reduced ability, compared to *hDIS3*, to rescue egg chamber degradation induced via induction of *Dis3^RNAi^* in somatic cells of the ovary. This contrasts with the ability of *hDIS3^H774Y^* to significantly rescue egg chamber degradation when Dis3 depletion was induced in the germline and may reflect functional differences in the two tissues or differing requirements in the levels of activity required in germline and somatic cells. This therefore suggests that human DIS3 may be more critical to somatic cell support of oocytes than the germline. However, further evidence is needed to affirm this postulation.

RNAi depletion alone gives no insight into the impact of the specific variant identified. Whether the identified missense variant destabilizes DIS3 and leads to a similar DIS3 depletion, is unknown. As observed in our work, *Dis3* RNAi KD can produce differing phenotypes under various GAL4 gene drivers even within the same tissue type (*nanos*GAL4 vs *Traffic-j*a*m* GAL4). Therefore, conclusions made on RNAi analysis alone allows for potentially egregious over/under assertions of pathogenicity for variants to be made. This can have a significant impact on patients suffering from POI and could lead to an incorrect causative gene being identified.

The variant c.2320C > T observed in our patient, when expressed in *Dis3-*depleted *Drosophila* under *Tj*-GAL4 substantially restored ovarian development. In contrast to Dis3 RNAi depleted *Drosophila* that had no visible ovarian structures, *hDIS*${3}^{p.H774Y}$ transgenic expression led to ovaries with normal gross morphology. When compared to the degree of rescue achieved by *hDIS3*, however, there were significant differences. This included aberrant polar cell arrangement in sequentially developing egg chambers as well as the presence of paired egg chambers. Paired egg chamber development is associated with failed germline cysts encapsulation by follicle cells. This observation is significantly increased in the presence of human variant H774Y *DIS3* when driven via Traffic-jam GAL4, a follicle cell driver. This is indicative of the role of the *DIS3* variant in the process of germline cyst encapsulation and, in turn, somatic support cells of the *Drosophila* ovary. These observations indicate that the c.2320C > T *DIS3* variant may be a hypomorphic allele. Such analysis is lacking for the previously reported *DIS3* variant and without such data, the impact of the variant remains unknown. Our data highlight the importance of *in vivo* variant modelling in the assessment of gene consequence.

### *DIS3* variants impact oocyte integrity and meiotic progression

DIS3 is a 3′-5′ catalytic exoribonuclease effector of RNA maturation and decay [32]. Post transcriptional modification and degradation of RNA species is considered critical to the modulation of gene expression and cell survival [84]. The function of DIS3 is well conserved and human DIS3 expression can complement Drosophila Dis3 depletion (Figure 5) or a point mutation in yeast *DIS3*, validating our use of these model organisms [85]. In somatic cells, DIS3 is specific to the nuclear RNA exosome, while DIS3L replaces it in the cytoplasmic exosome. DIS3 participates in the maturation of ribosomal RNAs from its precursor, as well as degradation of other RNAs, including PROMPTs and eRNAs. During development mammalian oocytes arrest at the first meiotic prophase and are transcriptionally inactive [86]. In this state there would be very little maturation and degradation of these RNAs. Instead, available RNA species in the oocyte cytoplasm predetermine the maternal transcriptome for subsequent embryonic development [87]. Whether DIS3 assumes a more cytoplasmic role in this situation is unknown. The modulation of the RNA transcriptome via RNA degradation pathways is therefore essential to oocyte development [34]. Aggregation of unwanted RNA transcripts, due to genetically perturbed RNA degradation machinery, are known to negatively impact meiosis, chromosome integrity, fertilization, zygotic gene activation and chromatin remodeling [34, 88, 89]. In mice depletion of *Dis3* in oocytes induces the accumulation of pervasive intergenic transcripts which is a result of both insufficient RNA degradation and defective transcription termination [34]. Together these changes prevent *Dis3* KO oocytes from resuming meiosis from their transcriptionally quiescent state. Failure of the oocyte to re-enter meiosis leads to a significant reduction in fertility for KO mice [34].

*Dis3* is considered critical to *Drosophila* multicellular development with temporal/spatial KD/KO resulting in complete loss of wing and ovarian structures and perturbed brain development [90, 91]. *Dis3* depleted ubiquitously in *Drosophila* is larval lethal at the L2 stage of development [90]. *Drosophila Dis3* participates in cell specific RNA turnover and metabolism both independently and in association with the RNA exosome [90]. In the ovary, conditional loss of exosome components, such as *Dis3*, result in premature germarium stage defects and a complete absence of the germline when driven under *nanos*GAL4 [92]. We similarly observed disrupted ovarian development in both germline and somatic cell *Dis3* KD.

Akin to mice, literature demonstrates that RNA degradation plays a key role in regulating *Drosophila* oocyte fate and inception [92]. Aberrant persistence of RNAs that escape degradation have been shown to induce a loss of oocyte maintenance and premature egg chamber death. Expression of patient variant, *DIS*${3}^{p.H774Y}$*,* in *Dis3*-depleted somatic cells of the *Drosophila* ovary via *Traffic-jam* GAL4 induces a significant degradation of developing egg chambers when compared to wild-type *hDIS3*. This egg chamber arrest and degradation occurs at the mid-oogenesis checkpoint of ovariole development. Mid-oogenesis egg chamber death is also observed in *Drosophila Pelota* mutants, defective in “no-go mRNA decay” and in *Twister* mutants, defective in a cytoplasmic RNA exosome cofactor. *Twister* mutants show oocyte defects in a germline context when driven via *nanos*GAL4 and no defects when driven somatically via *Traffic-jam* GAL4 [92]. However, it is noted that defects in other genes involved RNA degradation pathways have varying phenotypes in *Drosophila* depending on if they target nuclear or cytoplasmic RNAs [92]. For example, RNA degradation pathways involved in nuclear RNA degradation (*rRNA-processing 6* (*rrp6*) and *mRNA transport 4* (*mtr4*) or simultaneous nuclear and cytoplasmic RNA degradation, (*Dis3*) result in earlier germline defects or a complete loss of the germline when compared to cytoplasmic exclusive factors [92]. This indicates that it is the persistence of unwanted RNA species in such mutants that results in a loss of oocyte maintenance and egg chamber death [92]. This suggests that at mid-oogenesis high fidelity maintenance of the maternal contribution of nuclear RNAs into the developing oocyte is required for meiotic progression and successful oogenesis. The presence of our patient variant *DIS*${3}^{p.H774Y}$ perturbs this process in our *Drosophila* model*.*

Variants in *DIS3* have the potential to negatively impact the 3′-5′ exoribonuclease and RNA degradation activity of the basal subunit. This may allow for pervasive RNAs to be retained within a cell which, as demonstrated in literature, is unfavorable to oocyte maintenance and results in egg chamber death. This is because germ cells exist in a specialized somatic cell niche that supports germ cell growth and maturation [93]. The nurse cells in *Drosophila* transfer accumulated RNAs to support oocyte and embryonic development [93]. There is some evidence that reproductive support cells, such as follicle cells, may traffic RNAs to the germline, but further details need to be elucidated [93].

In mice, somatic cell cues are essential for translation in oocytes and the regulation of such is critical to embryonic development [94]. Therefore, if RNA degradation and regulation pathways are perturbed in somatic cells this has the potential to impact the functional niche and consequently the successful development of oocytes [94]. Our data supports the necessity of RNA regulation via *Dis3* in *Drosophila* ovarian somatic cell development and how the absence of Dis3 in somatic cells results in a complete loss of germ cells and ovarian structures.

The *DIS3* variant identified *DIS*${3}^{p.H774Y}$ in our patient negatively impacts egg chamber development supporting a potential pathogenic effect on RNA clearance in developing oocytes. These findings align with the presenting patient phenotype of secondary amenorrhea rather than complete loss of ovarian development and function (Table 1). Given the ubiquitous role of the RNA exosome in cellular function, it could be predicted that complete LoF could cause multisystem disease. Indeed, variants in *DIS3* and exosome components have been implicated in other diseases such as cancer [31, 32]. The isolated POI phenotype of the patient described in this study and the mild functional impact of the variant could reflect the sensitivity of the ovaries to damage. It is likely that the hypomorphic allele only partially disrupts DIS3 function and this disruption is enough to cause oocyte death but is tolerated by other organs and tissues.

Although the phenotype observed in our rescue flies is mild, our data are consistent with functional impairment and a hypomorphic allele. Whether this degree of impairment translates to human POI remains uncertain. Supporting the likelihood of *DIS3* being a bona fide “POI gene” is the fact that its disruption in multiple animal models leads to an ovarian phenotype and independent rare predicted-pathogenic *DIS3* variants have now been identified in two unrelated individuals with POI. These data highlight the critical role of the RNA exosome and its components in fertility and indicates that variants in *DIS3* should be considered a potential cause of POI.

## Supplementary Material

Kline_et_al_FIGURE_S1_ioae148

Kline_et_al_TABLE_S1_ioae148

## Data Availability

Restrictions apply to the availability of some, or all data generated or analysed during this study to preserve patient confidentiality or because they were used under license. The corresponding author will on request detail the restrictions and any conditions under which access to some data may be provided.
