# Supplementary material for: Functional characterization of human recessive DIS3 variants in premature ovarian insufficiency
Source: Biol Reprod. 2024 Oct 14;112(1):102–18. doi: 10.1093/biolre/ioae148 (PMC11736438; doi:10.1093/biolre/ioae148)
Supplement: Kline_et_al_FIGURE_S1_ioae148 [file kline_et_al_figure_s1_ioae148.pdf]

Functional characterisation of human recessive *DIS3* variants in premature ovarian insufficiency

Journal: Biology of Reproduction

Brianna L. Kline[1,2], Nicole A. Siddall[3], Fernando Wijaya[3], Luisa Orlando[4], Shabnam Bakhshalizadeh[1,2], Fateme Afkhami[5], Katrina M. Bell[1], Sylvie Jaillard[1,6,7] , Gorjana Robevska[1], Jocelyn A. van den Bergen[1], Shirin Shahbazi[5], Ambro van Hoof[4], Katie L. Ayers[1,2], Gary R. Hime[3], Andrew H. Sinclair[1,2], Elena J. Tucker[1,2]

1. Murdoch Children's Research Institute, Melbourne, Australia
2. Department of Paediatrics, University of Melbourne, Melbourne, Australia
3. Department of Anatomy and Physiology, University of Melbourne, Melbourne, Australia
4. Department of Microbiology and Molecular Genetics, University of Texas Health Science Centre at Houston, Houston, TX, USA
5. Department of Medical Genetics, Faculty of Medical Sciences, Tarbiat Modares University, Tehran, Iran
6. INSERM, Institut de Recherche en Santé, Environement et Travail, University of Rennes, Rennes, France
7. CHU Rennes, Service de Cytogénétique et Biologie Cellulaire, F-35033, Rennes, France

Corresponding author: Dr Elena J. Tucker elena.tucker@mcri.edu.au

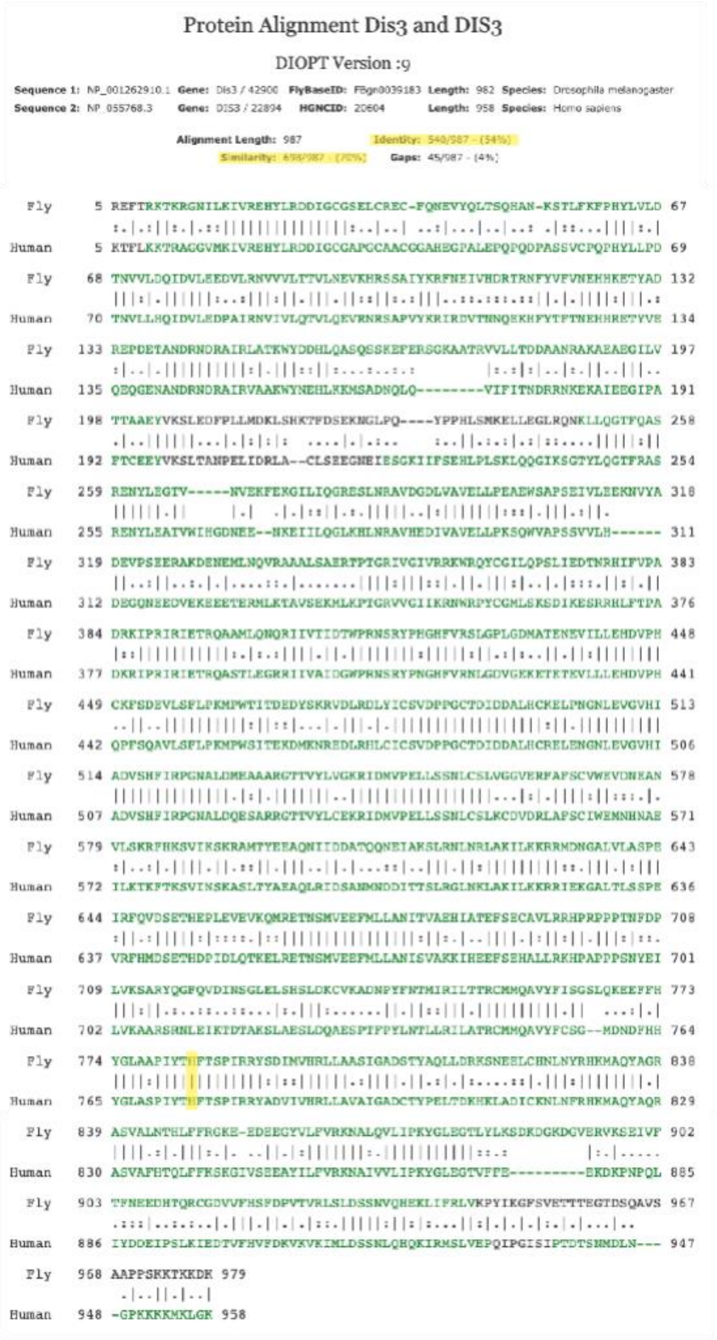

Supplementary Figure S1 Protein alignment of human *DIS3* and its orthologue, *Dis3*, in *Drosophila*. Dis3 shows 54% identity (lines) and 70% similarity (double dots) to the human DIS3 protein. Alignment demonstrates significant identity in surrounding residues to our residue of interest. The variant in our patient is in a residue (highlighted) that is identical at position 783 in *Drosophila*.
