# Supplementary material for: Functional characterization of human recessive DIS3 variants in premature ovarian insufficiency
Source: Biol Reprod. 2024 Oct 14;112(1):102–18. doi: 10.1093/biolre/ioae148 (PMC11736438; doi:10.1093/biolre/ioae148)
Supplement: Kline_et_al_TABLE_S1_ioae148 [file kline_et_al_table_s1_ioae148.pdf]

Functional characterisation of human recessive *DIS3* variants in premature ovarian insufficiency

Journal: Biology of Reproduction

Brianna L. Kline[1,2], Nicole A. Siddall[3], Fernando Wijaya[3], Luisa Orlando[4], Shabnam Bakhshalizadeh[1,2], Fateme Afkhami[5], Katrina M. Bell[1], Sylvie Jaillard[1,6,7] , Gorjana Robevska[1], Jocelyn A. van den Bergen[1], Shirin Shahbazi[5], Ambro van Hoof[4], Katie L. Ayers[1,2], Gary R. Hime[3], Andrew H. Sinclair[1,2], Elena J. Tucker[1,2]

1. Murdoch Children's Research Institute, Melbourne, Australia
2. Department of Paediatrics, University of Melbourne, Melbourne, Australia
3. Department of Anatomy and Physiology, University of Melbourne, Melbourne, Australia
4. Department of Microbiology and Molecular Genetics, University of Texas Health Science Centre at Houston, Houston, TX, USA
5. Department of Medical Genetics, Faculty of Medical Sciences, Tarbiat Modares University, Tehran, Iran
6. INSERM, Institut de Recherche en Santé, Environement et Travail, University of Rennes, Rennes, France
7. CHU Rennes, Service de Cytogénétique et Biologie Cellulaire, F-35033, Rennes, France

Corresponding author: Dr Elena J. Tucker elena.tucker@mcri.edu.au

Table S1 Pathogenicity predications for WES identified gene variants

Gene	NM	gnomad Genomes	Function	Variant	gDNA (hg38)	cDNA	Protein	PolyPhen	SIFT	Mutation Taster	CADD	Other
DIS3	NM_001128226.3	0	RNA surveillance, processing, and degradation	Homozygous missense	G>A	c.2320C>T	p.(His774Tyr)	Probably Damaging (0.999)	Damaging	Disease causing	29.6	*Dis3* depletion reduces female mouse fertility [1]
MTF1	NM_005955.3	3.19 x 10^{-5}	Zinc dependent transcriptional regulator	Missense	G>A	c.1933C>T	p.(Arg645Trp)	Benign (0.001)	Damaging	Disease causing	25.6	Upregulation of human *MTF1* associated with ovarian tumours [2]. Truncation of *Drosophila Mtf-1* severely compromises female fly fertility [3]
		0		Missense	G>C	c.299C>G	p.(Pro100Arg)	Possibly damaging (0.806)	Damaging	Disease causing	32	
RAD51B	NM_001321809.2	3.19 x 10^{-5}	Homologous recombination repair	Homozygous missense	A>G	c.1198A>G	p.(Thr400Ala)		Damaging	Polymorphism	0.771	Missense and Loss of function variants associated with breast and ovarian cancer risk [4]
APC2	NM_001351273.1	0	Transcriptional regulator	Homozygous missense	C>T	c.4403C>T	p.(Pro1468Leu)	Benign (0.242)	Damaging	Disease causing	23.1	Colon cancer tumour suppressor, linked to intellectual disability phenotypes [5, 6]. Deficiency associated with subfertility in female mice [7]
MGA	NM_001080541.3	0	Dual specificity transcription factor	Homozygous missense	G>T	c.9004G>T	p.(Asp3002Tyr)		Damaging	Disease causing	25.9	Loss of function mutations associated with lung, ovarian and colon cancer [8-11].

1. Wu, D. and J. Dean, *Reduced female fertility due to sequestration of RNA Pol II by pervasive transcription in exosome RNase-depleted oocytes.* Cell Rep, 2023. **42**(10): p. 113247.
2. Ji, L., et al., *Knockout of MTF1 Inhibits the Epithelial to Mesenchymal Transition in Ovarian Cancer Cells.* J Cancer, 2018. **9**(24): p. 4578-4585.
3. Gunther, V., et al., *Dissection of Drosophila MTF-1 reveals a domain for differential target gene activation upon copper overload vs. copper starvation.* Int J Biochem Cell Biol, 2012. **44**(2): p. 404-11.
4. Golmard, L., et al., *Germline mutation in the RAD51B gene confers predisposition to breast cancer.* BMC Cancer, 2013. **13**: p. 484.
5. Polakis, P., *The adenomatous polyposis coli (APC) tumor suppressor.* Biochim Biophys Acta, 1997. **1332**(3): p. F127-47.
6. Almuriekhi, M., et al., *Loss-of-Function Mutation in APC2 Causes Sotos Syndrome Features.* Cell Rep, 2015. **10**(9): p. 1585-1598.
7. Mohamed, N.E., et al., *APC2 is critical for ovarian WNT signalling control, fertility and tumour suppression.* BMC Cancer, 2019. **19**(1): p. 677.
8. Mathsyaraja, H., et al., *Loss of MGA repression mediated by an atypical polycomb complex promotes tumor progression and invasiveness.* Elife, 2021. **10**.
9. Weberpals, J.I., et al., *Tumor genomic, transcriptomic, and immune profiling characterizes differential response to first-line platinum chemotherapy in high grade serous ovarian cancer.* Cancer Med, 2021. **10**(9): p. 3045-3058.
10. Jo, Y.S., et al., *Somatic mutation of a candidate tumour suppressor MGA gene and its mutational heterogeneity in colorectal cancers.* Pathology, 2016. **48**(5): p. 525-7.
11. Cancer Genome Atlas Research, N., *Comprehensive molecular profiling of lung adenocarcinoma.* Nature, 2014. **511**(7511): p. 543-50.
